# Chlorotoxin and Lung Cancer: A Targeting Perspective for Drug Delivery

**DOI:** 10.3390/pharmaceutics14122613

**Published:** 2022-11-26

**Authors:** Archana Shrestha, Behnaz Lahooti, Constantinos M. Mikelis, George Mattheolabakis

**Affiliations:** 1 School of Basic Pharmaceutical and Toxicological Sciences, College of Pharmacy, University of Louisiana at Monroe, Monroe, LA 71209, USA; 2 Department of Pharmaceutical Sciences, School of Pharmacy, Texas Tech University Health Sciences Center, Amarillo, TX 79106, USA; 3 Laboratory of Molecular Pharmacology, Department of Pharmacy, University of Patras, 26504 Patras, Greece

**Keywords:** nanoparticles, chlorotoxin, lung cancer, active targeting, cytotoxicity, morphometric analysis

## Abstract

In the generational evolution of nano-based drug delivery carriers, active targeting has been a major milestone for improved and selective drug accumulation in tissues and cell types beyond the existing passive targeting capabilities. Among the various active targeting moieties, chlorotoxin, a peptide extracted from scorpions, demonstrated promising tumor cell accumulation and selection. With lung cancer being among the leading diagnoses of cancer-related deaths in both men and women, novel therapeutic methodologies utilizing nanotechnology for drug delivery emerged. Given chlorotoxin’s promising biological activity, we explore its potential against lung cancer and its utilization for active targeting against this cancer’s tumor cells. Our analysis indicates that despite the extensive chlorotoxin’s research against glioblastoma, lung cancer research with the molecule has been limited, despite some promising early results.

## 1. Introduction

The advent of nanotechnology presented innovative solutions for drug delivery to the existing traditional methodologies. Numerous new nanosized delivery carriers provided novel perspectives and opportunities to overcome traditional drug delivery limitations. At the same time, a stream of pioneering variations on these nanocarrier designs expanded their original capabilities [1]. For example, liposomes evolved from basic lipid bilayers to long-circulating liposomes and immunoliposomes [2], while similar “evolutionary steps” occurred in virtually every other drug delivery nanocarrier type [2,3,4,5,6].

Active targeting methodologies were introduced for the selective accumulation of drugs to a subset of cells in vivo, in addition to any passive drug accumulation to the desired tissues that may take place [1]. This straightforward but challenging objective relies on molecules that have an affinity towards specific proteins. Certain proteins/receptors are overexpressed on diseased vs. normal cells, and using molecules to identify and bind to these proteins can accommodate the specific delivery of compounds to these cells [7]. Antibodies, antibody fragments, aptamers, sugars, and peptides have prominently been used for their ability to identify, bind, and promote endocytosis of drug carriers attached to them preferentially to specific cells [7,8]. Active targeting found applicability to various drug carrier types, including liposomes (i.e., immunoliposomes) [2], polymeric nanoparticles [9], dendrimers [10], and inorganic nanoparticles [11], among others. Briefly, active targeting has been used to target cells of the immune system [12,13], endo-/epithelial cells [14,15], and, most prominently, cancer cells [7]. 

Among the different molecule types used for active targeting, peptides have attracted extensive interest [16,17]. By using peptides conjugated with drugs or carriers for targeting receptors overexpressed in diseased cells, active targeting against these cells could be achieved [18]. Receptor-specific ligands based on peptides present advantages compared to antibodies or small molecules. These include: (a) decreased immunogenicity; (b) controlled chemistry capable of producing a large variety of distinct potential structures; and (c) the possibility of reduced peptide production cost, depending on the length and complexity of the peptide [19]. Representative of the peptide structural variety for active targeting is that peptides as short as three amino acids in length have been used for active targeting of drug delivery carriers [20], while much larger peptides have also been evaluated [19].

Animal venoms are complex mixtures of bioactive proteins and peptides, among other compounds, naturally designed (or naturally selected) to have substantial effects at low concentrations on a potential recipient [21,22]. In this article, we focus on an animal venom-derived peptide known as chlorotoxin (CTX), which is increasingly recognized for its capacity to preferentially attach to cancer cells and is used for active targeting. Although we provide a brief overview of how chlorotoxin came to prominence for differentiating glioma cancer cells vs. normal cells, we focus on its use in lung cancer and describe the up-to-date literature on this cancer type. With lung cancer being the second most frequently observed cancer in new cases and the leading cause of cancer-related deaths in the USA, active targeting methodologies can potentially enhance the therapeutic abilities of drugs against the disease [23]. CTX’s demonstrated potential for active targeting should be of significant value against this cancer type. Here, we show that early research on CTX presented a favorable behavior for active targeting against cancer cells, including lung cancer. However, we identified a rather contrasting finding, where there is limited literature on CTX’s activity against lung cancer cells decades after the molecule’s discovery and its recognition for active targeting. As the reasons for CTX’s limited applicability in lung cancer are not apparent, we attempt to build a comprehensive understanding of its relatively limited application for active targeting against this disease.

## 2. Materials and Methods

### 2.1. Materials

DMEM and F12K media were purchased from GibcoTM (Life technologies, Carlsbad, CA, USA). Bovine Serum Albumin (BSA) was obtained by R&D Systems (Minneapolis, MN, USA). CellTiter-Fluor cell viability Assay was obtained from Promega (WI, USA). Other chemicals were of analytical grade, obtained from Fisher, VWR or Sigma. Recombinant Chlorotoxin was obtained from Alfa chemistry (#ACM163515353; Ronkokoma, NY, USA). 

### 2.2. Cell Culture

A549 cells were cultured in DMEM/F12K media, with 10% fetal bovine serum and 1% penicillin/streptomycin. H1975 cells were cultured in RPMI media supplemented with 10% fetal bovine serum and 1% penicillin/streptomycin. Both cell lines were maintained at 37 °C with 5% CO_2_ supply in humidified conditions. 

### 2.3. Cell Viability

Briefly, 1 × 10^4^ cells of A549 or H1975 were seeded in the wells of a 96-well, black, optical bottom plate with complete media. Following overnight incubation at 37 °C, the cells were treated with varying concentrations of CTX in DMSO diluted into the media, and incubated for 24, 48, and 72 h. Each concentration was evaluated in sextuplicates. An equal volume of DMSO in complete media served as the control. Following incubation, live cells were detected using the CellTiter-Fluor cell viability Assay, following manufacturer’s instructions, and predicted IC50 values were determined using GraphPad Prism (Version 8, GraphPad Software LLC, San Diego, CA, USA). 

### 2.4. Quantitative Phase Imaging Microscopy

We seeded 25,000 cells/well for A549 and 30,000 cells/well for H1975 in 24-well glass bottom plates (Cellvis Cat#P24-1.5H-N), overnight at 37 °C. Subsequently, varying concentrations of CTX in DMSO diluted in media were added to wells in triplicates, and the cells were under constant observation for 24 h using Livecyte (Phasefocus, Sheffield, UK) for determining doubling time and quantifying morphological characteristics. Control group was an equal volume of DMSO in media. 

### 2.5. Statstical Analysis

We performed one-way ANOVA followed by post hoc Tukey’s test to determine the significance of difference between the treated vs. untreated groups unless otherwise specified. We present the mean values ± standard errors; *p* values < 0.05 were considered statistically significant.

## 3. Discussion 

### 3.1. Chlorotoxin Selectively Binds to Cancer Cells—Receptor Targeting

**Figure 1:**
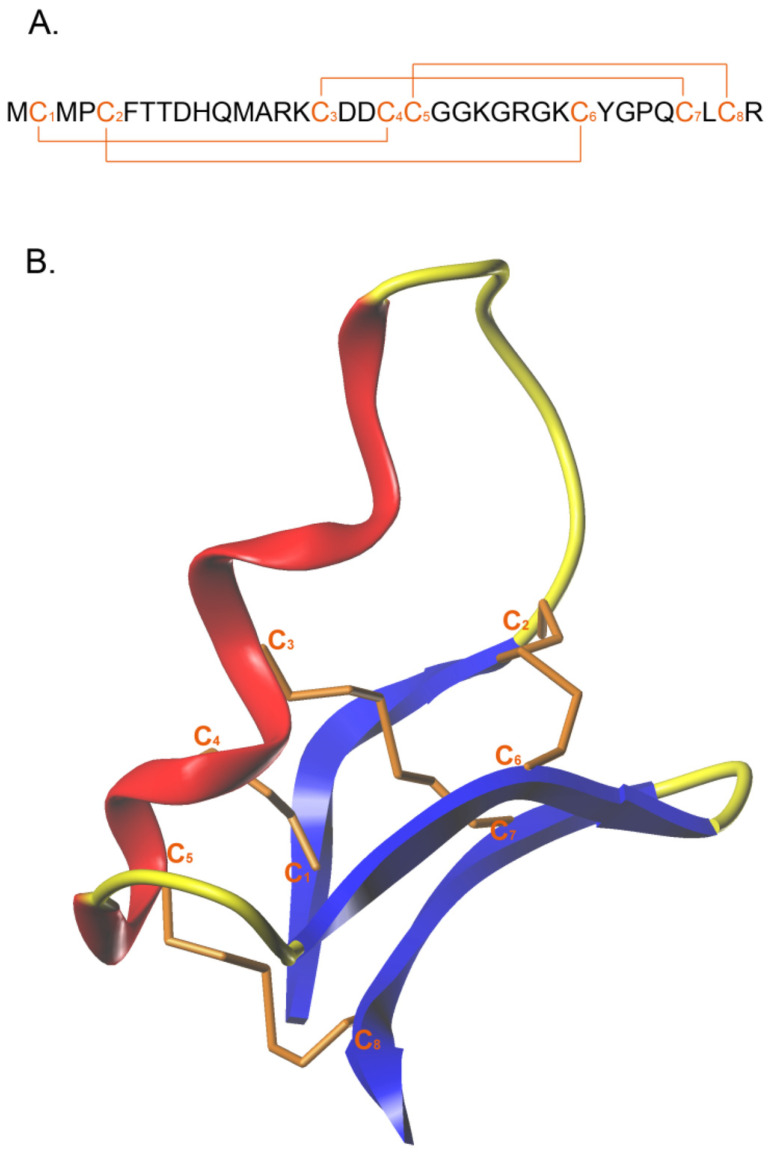
Chlorotoxin’s amino acid sequence (**A**) and structure (**B**) with the disulfide bonds presented. Reprinted with permission from Ref. [25], 2015, MDPI.

CTX is a neurotoxin with 36 amino acids and four disulfide bonds between the cysteine residues at 2 to 19, 5 to 28, 16 to 33, and 20 to 35 (Figure 1) [24]. The molecule was isolated from the *Leiurus quinquestriatus* scorpion venom [25] and attracted attention for its capacity to differentiate between glioma cancer cells and normal cells, favored by CTX’s apparent/hypothesized capacity to cross the blood-brain barrier [26]. The list of potential cancer cell targets was subsequently expanded further. 

Two conjugates of CTX, one with ^131^I [27], a radioactive iodine, and the other with Cy5.5 [28], a NIR fluorescent molecule, were synthesized, and they presented the potential for identification and visual discrimination of cancerous vs. normal tissue. The studies revealed that CTX’s tumor cell discriminating properties in vivo could improve pre-operative tumor identification and identify and mark tumor areas with a visually detectable dye during surgical procedures. Though the focus was on glioma, the technology also presented high potential against medulloblastoma, prostate cancer, intestinal cancers, and sarcoma. Hence, this approach would permit improved tumor detection and surgical precision, achieving higher precision in removing tumor foci and, thus, minimizing the damage to normal tissue [28]. Furthermore, these applications illustrated CTX’s potential for drug delivery. Simply stated, since CTX can carry and deliver a dye molecule to the tumor cells, it can potentially deliver other types of molecules, such as drugs.

CTX binds to small-conductance epithelial chloride channels, inducing cellular internalization [29]. Representatively, as glioma cells reduce in size, through reduction of the cytoplasm volume, this requires secretion of K^+^ and Cl^−^ ions, among others [29]. The result is an increase of chloride channel abundance to support the process in the glioma cells, while these chloride channels are absent or with a relatively lowered presence on normal cells. Thus, CTX’s chloride channel targeting favors the molecule’s attachment on glioma cells. Further analysis indicated that CTX also selectively binds to matrix metalloproteinase- (MMP)-2 [26]. This receptor is of particular interest as it participates in the degradation of the extracellular matrix and associates with increased metastatic potential for different tumor cells [30,31]. *MMP*-2 upregulation has been detected in melanoma [32], breast [33,34], ovarian [35,36], pancreatic [37,38], prostate [39,40], and lung cancers [31,41], among others, and associates with metastasis or poor prognosis. 

Finally, Kesavan et al. [42] reported that CTX also binds to Annexin A2. They utilized the ^131^I-CTX complex, which attached to PANC-1 cells in an annexin A2-expression dependency. Further analysis in HUVECs revealed the binding of CTX to Annexin A2. In another study, McGonigle et al. [43] reported that CTX targeted Neuropilin-1, a tumor, and endothelial cell endocytic receptor. This plurality of potential tumor cell-overexpressed receptor targets for CTX presents the current ambiguity in our understanding of its function, though the conclusion that CTX preferentially targets cancer over normal cells remains. 

### 3.2. Chlorotoxin and Lung Cancer: Promising but Limited Research

With the elevated lung cancer occurrence and mortality rate, it is evident that novel methodologies for improved therapeutics are necessary. Active targeting against lung cancer cells, similar to other cancers, presents potential advantages for improved pharmacokinetics, reduced side effects, and improved therapeutic outcomes. As described above, CTX demonstrated a promising capacity to differentiate between tumor and normal cells, which prompted its evaluation for active targeting against lung cancer cells, as presented below.

Lyons et al. [44] used a biotin conjugate of a synthetic, biologically active CTX to evaluate its capacity to attach to different cancer types. Among the cancer types tested, the authors evaluated lung carcinomas and reported that the molecule demonstrated a consistent positive cancer tissue staining (i.e., small cell lung carcinomas), while presenting a negative staining to normal tissues, including lung tissue. 

In the study using the Cy5.5-CTX conjugate mentioned above, Veiseh et al. [28] reported that they were able to detect lung metastasis in an animal model of prostate cancer following intravenous (i.v.) administration of the conjugate using transgenic mice that expressed SV40T gene at the prostate epithelium. Gribbin et al. [45] reported that a phase I clinical trial of the ^131^I-CTX molecule, also termed ^131^I-TM-601, demonstrated tumor-specific uptake, following i.v. administration and observation with a gamma camera or SPECT imaging. Among the patients with different cancer types that presented a positive tumor uptake by the CTX-modified molecule, there were also patients with non-small cell lung cancer. Contrastingly, in patients with small cell lung cancer, tumor uptake of the molecule was not detected. Kesavan et al. [42] reported a strong binding affinity of CTX with A549 cells, which was later corroborated by Wiranowska et al. [46], who demonstrated that TM-601 was taken up by A549 lung cancer cells in vitro. Thus, these studies corroborate a strong potential for CTX to be used for lung cancer active targeting.

Not surprisingly, CTX was evaluated for active drug delivery. In an approach that resembles the ^131^I-CTX and Cy5.5-CTX conjugates, Graf et al. [47] developed a Pt(IV) complex with CTX. Platinum (Pt)-based compounds are at the forefront of lung cancer treatment [48]. Pt is the central metal atom in the Pt(II) coordinated complexes in drugs such as cisplatin, oxaliplatin, carboplatin, and others [49], while Pt(IV) drugs have been produced, as Pt(IV) is reduced to the active Pt(II) in situ in the cells [50]. The researchers determined that the CTX-Pt (i.e., Pt(IV)) presented relatively decreased cytotoxicity in A549 lung cancer cells compared to cisplatin (i.e., Pt(II)). The authors reported that this was also previously observed with other ligand-Pt(IV) conjugates, such as with cell-penetrating peptides, attributed to the Pt(IV)’s inherent reduced toxicity compared to cisplatin (i.e., Pt(II)). Nonetheless, by including additional cancer cells in their study, the authors concluded that the CTX conjugate with Pt(IV) had higher toxicity vs. Pt(IV) alone, attributed to an active targeting effect as the effect was cell type-dependent, i.e., receptor expression-mediated [47]. 

More recently, Yang et al. [51] reported the development of a magnetic nanochain linked to CTX and conjugated with curcumin. The researchers indicated that the CTX-conjugate targeted A549 cells in vitro, and the tumors were detectable using magnetic resonance imaging in vivo. Finally, daily i.v. administration of the conjugate to the animals inhibited early tumor growth.

With a focus on nanotechnology, but not specifically on lung cancer, Qin et al. [52] developed a CTX-modified liposomal formulation of doxorubicin. The authors evaluated the nanocarrier’s capacity to target metastatic breast tumors that develop in the lungs, using mice bearing highly metastatic 4T1 cell tumors. Compared to non-modified liposomes, the CTX-modified liposomes had a more robust uptake by the 4T1 cells in vitro and presented a stronger accumulation in the tumor area in vivo. Furthermore, the CTX-modified liposomes inhibited both primary tumor growth and lung metastases development more potently than the unmodified liposomes. Briefly, no metastatic lung nodules were detected in the CTX-modified liposomes treated group, while the unmodified liposomes treated group presented a 4/6 incidence rate of metastasis in the lungs.

Although these studies demonstrated a promising potential of CTX for lung cancer treatment, the research on the topic has not expanded. This becomes more intriguing by recognizing that: (a) identification of CTX’s binding to chloride channels took place in 1993 [53]; (b) CTX active targeting against tumor methodologies developed as early as 1998 [27]; and (c) CTX active targeting against lung cancer were reported as early as 2002 [44]. These dates indicate a more than two decades old field. For comparison, three other peptides, Arg-Gly-Asp (RGD), HER-2, and TAT peptides with similarly ~2–3 decades since their discovery [54,55,56], have been extensively studied for active targeting against lung cancer. For example, limited research (~10 articles) has been produced for CTX and lung cancer vs. hundreds of articles for the other three peptides. Although this analysis may not be comprehensive, it is apparent that chlorotoxin utilization in lung cancer has been limited. In contrast, a search on CTX and glioma presents a different perspective, with >110 articles on the topic, and relatively similar numbers of papers for the other three aforementioned targeting moieties on the same cancer. 

It seems that early research on CTX and its tumor-targeting capacity was dominated by glioma-focused studies, which could have contributed to the high CTX-based publications on this disease. Nonetheless, the skewed result of limited CTX’s research abundance against lung cancer does not necessarily indicate a limited activity of the molecule against this cancer type or any other cancer, and the presented research above could indicate the contrary.

### 3.3. Identification and Comparison of CTX’s Targeting Capacity

The limited but promising data on CTX and lung cancer draws a favorable picture of the molecule’s potential against this disease, but does not fully address the CTX’s importance for active targeting against lung cancer. A comparison between CTX and other targeting moieties, or a comparison of targeted receptor expressions, could potentially assist in further evaluating the molecule’s potential for targeting lung cancer cells. Unfortunately, we did not identify literature comparing CTX and other targeting moieties directly. Furthermore, expression levels of different receptors do not lead to an ideal comparison. The reason is that, at least in the studies we evaluated during the preparation of this manuscript, the researchers describe a relative receptor expression between tumor and normal tissue/cells and not an absolute quantification of receptor abundance per cell or gram of tissue or similar. An interesting indicator for absolute quantification would be the analysis of RNA-seq data, where normalized FPKM values for genes are provided, and, by extension, the expression could be obtained for different genes based on quantitative values. Unfortunately, as RNA levels to protein expression correlation does not necessarily take place, the FPKM values do not ensure that they correlate to the corresponding protein expressions or even more, to membrane-bound receptors [57,58]. Furthermore, the tissue expression of specific proteins may not necessarily correspond to tumor-(or tumor cell-) specific protein expression, as the baseline expression of a receptor in normal cells may vary by cell type, organ, and other complex physiological or gene regulatory mechanisms [57,58]. In vitro cell lines can be viable alternative models but do not necessarily reflect the in vivo tumor microenvironment [59]. 

Another perspective is the binding constant of the targeting moiety to the targeted receptors. For example, two potential binding sites were identified for CTX against glioma cells, a high-affinity site with a dissociation constant (Kd) = 4.2 nM and a low-affinity site with a Kd = 660 nM. However, it was assumed that the targets are glioma-specific chloride ion channels or a receptor that modulates chloride channel’s activity [27]. In another study, de-amidated CTX, which is assumed to be generated in situ at the tumors following CTX administration, demonstrated binding with Neuropilin-1 with a Kd value of ~240 nM [43]. Deshane et al. [60] evaluated CTX’s binding to MMP-2 using an enzyme-linked immunosorbent-based assay and identified 50% CTX binding was achieved at a concentration of 115 nM. For comparison, two FDA-approved antibodies, Cetuximab, a monoclonal antibody against EGFR for treatment of various cancers, has a Kd of 0.38 nM [61], and Trastuzumab (i.e., herceptin), a recombinant antibody against HER2, has a Kd of ~5–9 nM [62]. In another case, folic acid, a molecule frequently used for active targeting against lung cancer, has a Kd value of ~0.4 nM [63,64]. Table 1 presents the Kd values for some representative molecules used in drug delivery for active targeting. Thus, CTX presents a relatively comparable, though potentially higher, Kd value compared to other targeting moieties used for active targeting in drug delivery. In fact, CTX’s binding capacity, as defined by its Kd values, should be characterized as medium strength affinity [65], similar to other peptides presented in Table 1. 

Although this information may indicate how CTX has the potential to target specific receptors, the actual tumor-type dependent abundance of receptors on tumor cells may produce variations to the molecule’s attachment capacity. A review by Dardevet et al. [30] reported that CTX was successfully used for dying lung cancer tissues vs. normal lungs, indicating successful selectivity. Furthermore, Kesavan et al.’s [42] comparison of CTX’s binding between A549 lung cancer cells and U87-MG glioblastoma cells demonstrated that the molecule should have comparable behavior in the two cancer types. Briefly, the authors quantified the binding of TM-601 (i.e., CTX) by measuring the radioactivity of technetium-99m-labeled TM-601 bound to glioblastoma cells (i.e., U87-MG), NSCLC cells (i.e., A549) and pancreatic cancer cells (i.e., Panc-1). The authors reported that A549 and U87-MG cells demonstrated comparable IC50 values (18.2 nM vs. 10.7 nM) while Panc-1 cells presented higher IC50 value (106 nM). Kesavan et al.’s results may indicate that CTX’s attachment capacity to lung cancer cells may be comparable to glioblastoma cells. However, it is challenging to generalize and reach the same hypothetical conclusion for all lung cancer types, as the study took place with a single lung cancer cell line. 

Similar to other peptides, CTX has limitations that may deter potential research on the molecule. For example, peptides have been limited by chemical and physical instability, short half-life, and challenges associated with cell membrane permeability or endosomal escape [73,74]. Furthermore, the CTX’s relatively long 36-aminoacid chain with four disulfide bonds and a 3D structure is not easily produced using traditional peptide synthesis. These factors can potentially become prohibitive for active research, which may explain any hesitation from the research community to rapidly expand in the lung cancer field. Yet, these limitations do not justify CTX’s limited utilization in lung cancer compared to glioblastoma, or in one cancer type vs. another.

### 3.4. CTX Activity on Lung Cancer Cells

The limited literature of CTX’s activity on lung cancer does not only hinder our understanding on the molecule’s potential as an active targeting agent, but also does not provide significant information on the activity of CTX directly to the lung cancer cells. For example, in an interesting study by Ayed et al. [75], the authors reported the effect of CTX’s C-terminal amidation on tumor cell proliferation/cytotoxicity using a variety of cell lines, including U87MG glioblastoma cells, MCF-7 breast cancer cells, PC3 prostate cancer cells, and A549 lung cancer cells. The overall conclusion of their study was that CTX did not demonstrate significant toxicity or inhibition of proliferation in these cell lines at 10 μM concentration up to 48 h. 

**Figure 2:**
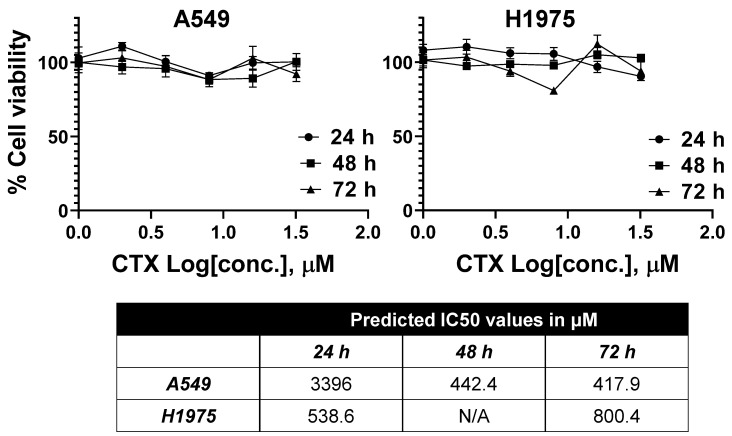
Cytotoxicity analysis of CTX at varying concentrations in A549 (**left**) and H1975 (**right**) cells at 24, 48, and 72 h. Different amounts of CTX were dissolved in an equal volume of DMSO, prior to dilution into complete media for each well to the targeted desired concentration, in sextuplicates. An equal volume of DMSO in complete media served as the control. All values are presented as averages with SEM. N/A: not available, i.e., IC50 value is unpredictable. The A549 and H1975 cell lines were obtained from ATCC.

**Figure 3:**
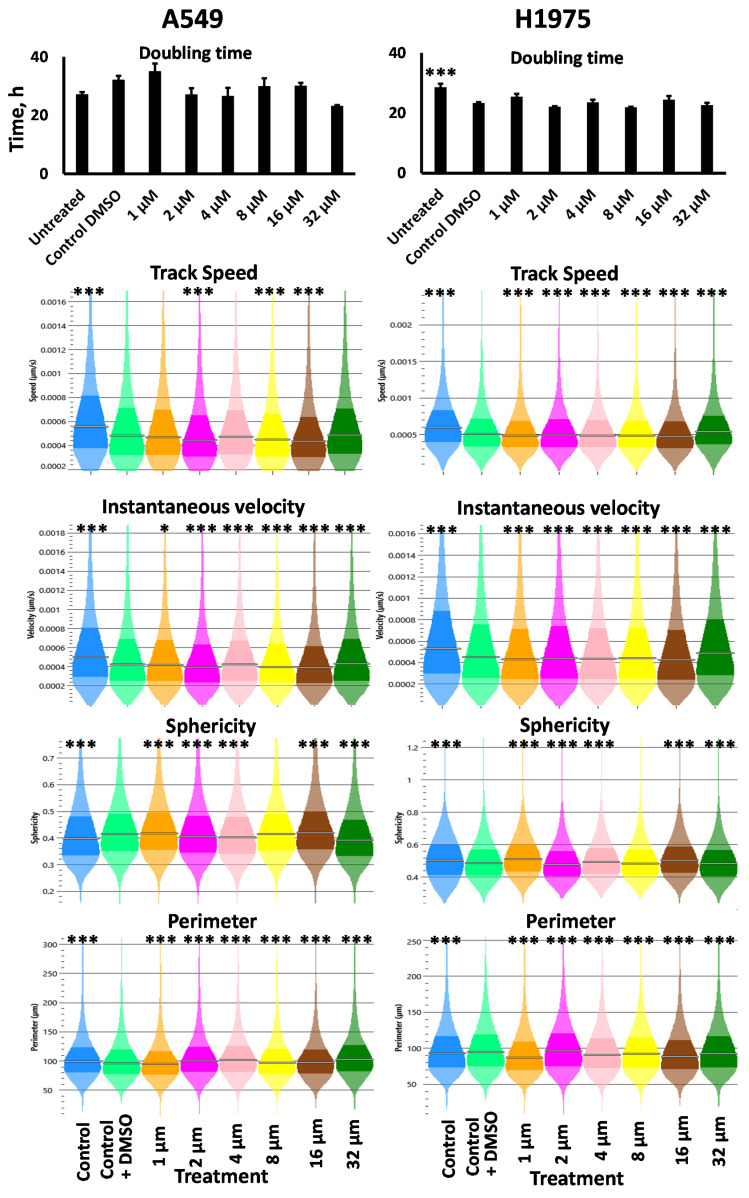
Cell doubling time, track speed, instantaneous velocity, sphericity, and perimeter were monitored for approximately 24 h using Livecyte QPI microscopy. A549 (**left**) and H1975 (**right**) cells were cultured in 24-well plates, and CTX at varying concentrations was introduced (similar to cytotoxicity analysis above). All samples were in triplicate wells. Cell doubling time data are the average of three wells per treatment group, with standard error of the mean. Colored graphs indicate value distribution based on values from all individually measured cells. The statistical analysis is one-way ANOVA, followed by Tukey’s post-hoc test. All statistical analyses reported are in comparison to the control-DMSO group. *: *p* < 0.05; ***: *p* < 0.001.

CTX’s promising potential as an active targeting moiety against lung cancer prompted us to evaluate the molecule against lung cancer cells. Therefore, we evaluated CTX’s activity against two lung cancer cell lines, A549 and H1975. First, we evaluated the cytotoxicity of the molecule in the two cell lines in a range of concentrations. Our analysis indicated that CTX did not demonstrate a significant cytotoxicity, with predicted IC50 values above its reported Kd binding concentrations (Figure 2). Similarly, we performed morphometric analyses via Quantitative Phase Imaging (QPI) microscopy, using a Livecyte microscope (Phase Focus, Sheffield, UK) [76], to evaluate the effect of various CTX concentrations on lung cancer cells for approximately 24 h (Figure 3). CTX did not significantly alter the doubling time for the two cell lines and did not cause an identifiable trend in the change of speed and instantaneous velocity for both cell lines, despite the statistical analysis indicating significant differences between concentrations. In fact, the statistical significances can be attributed to the large number of cells being evaluated simultaneously by the microscope, which allows for increased accuracy in the detected properties per study group. Nonetheless, the increasing CTX concentration did not demonstrate a precise behavior in the parameters mentioned above. Finally, increasing CTX’s concentration did not present any identifiable trend regarding the cells’ sphericity and perimeter as well. These results indicate that in the studied concentrations, CTX did not induce appreciable morphometric changes in the cells [76]. Thus, we did not identify an impact on the lung cancer cells’ activity by CTX (for the tested parameters), at least in concentrations up to 32 μM, which are higher than the molecule’s suggested Kd value. 

## 4. Conclusions

CTX appears to have no appreciable effect directly on cancer cells, which can contribute to its classification as purely a targeting moiety. With the existing body of literature, there is no reason to assume that CTX does not work for active targeting against lung cancer cells. Furthermore, the existing limited research indicates the opposite, and that CTX can be a useful targeting moiety against the disease. Thus, the molecule’s limited utilization against lung cancer may only be limited by the lack of active research focus on the topic until now. Nonetheless, further work is necessary to ascertain its potential for active targeting against this disease and the potential benefits that it can provide, similar to the benefits that the molecule provided against glioblastoma. 

## Figures and Tables

**Table 1:** Representative active targeting moieties and their respective dissociation constant values. The list includes peptides, antibodies, and aptamers used for active targeting.

Targeting Moiety	Molecule Type	Target	Kd (nM)	Ref.
GE11	Peptide	EGFR	22.28	[66]
QRHKPRE	Peptide	EGFR	50	[67]
RGD	Peptide	Integrins α_ν_β_3_ and α_ν_β_5_	3.2–100	[68]
Trastuzumab	Antibody	HER2	5–9	[62]
Bevacizumab	Antibody	VEGF	0.5	[68]
NOX-E36	Aptamer	MCP-1	1.32	[69]
NU172	Aptamer	Thrombin	0.3–0.5	[69]
AB3	Aptamer	OFA/iLRP	101	[69]
AO-01	Aptamer	Matrix-binding domain	180	[70]
Hyaluronic acid	Poly saccharide	CD44	0.3	[71]
Transferrin	Glycoprotein	Transferrin Receptor	0.23	[72]

## Data Availability

Data available on request.
